# Lifestyle and Socioeconomic Determinants of Multimorbidity Patterns among Mid-Aged Women: A Longitudinal Study

**DOI:** 10.1371/journal.pone.0156804

**Published:** 2016-06-03

**Authors:** Caroline A. Jackson, Annette J. Dobson, Leigh R. Tooth, Gita D. Mishra

**Affiliations:** 1 Centre for Longitudinal and Life course Research, School of Population Health, University of Queensland, Herston Road, Herston, QLD 4006,Australia; 2 Usher Institute of Population Health Sciences and Informatics, No. 9 Edinburgh Bioquarter, 9 Little France Road, Edinburgh, EH16 4UX, United Kingdom; Mayo Clinic, UNITED STATES

## Abstract

**Background:**

Little is known about patterns of associative multimorbidity and their aetiology.

We aimed to identify patterns of associative multimorbidity among mid-aged women and the lifestyle and socioeconomic factors associated with their development.

**Methods:**

Participants were from the Australian Longitudinal Study on Women’s Health. We included 4896 women born 1946–51, without multimorbidity in 1998. We identified multimorbidity patterns at survey 6 (2010) using factor analysis, and related these patterns to baseline lifestyle and socioeconomic factors using logistic regression. We dichotomised factor scores and determined odds ratios (ORs) with 95% confidence intervals (CIs) for associations between characteristics and odds of a high versus low factor score.

**Results:**

We identified five multimorbidity patterns: psychosomatic; musculoskeletal; cardiometabolic; cancer; and respiratory. Overweight and obesity were respectively associated with increased odds of having a high score for the musculoskeletal (adjusted ORs 1.45 [95% CI 1.23, 1.70] and 2.14 [95% CI 1.75, 2.60]) and cardiometabolic (adjusted ORs 1.53 [95% CI 1.31, 1.79] and 2.46 [95% CI 2.02, 2.98]) patterns. Physical inactivity was associated with increased odds of a high score for the psychosomatic, musculoskeletal and cancer patterns (adjusted ORs 1.41 [95% CI 1.13, 1.76]; 1.39 [95% CI 1.11, 1.74]; and 1.35 [95% CI 1.08, 1.69]). Smoking was associated with increased odds of a high score for the respiratory pattern. Education and ability to manage on income were associated with increased odds of a high score for the psychosomatic pattern (adjusted ORs 1.34 [95% CI 1.03, 1.75] and 1.73 [95% CI 1.37, 1.28], respectively) and musculoskeletal pattern (adjusted ORs 1.43 [95% CI 1.10, 1.87] and 1.38 [1.09, 1.75], respectively).

**Conclusions:**

Distinct multimorbidity patterns can be identified among mid-aged women. Social inequality, physical activity and BMI are risk factors common to multiple patterns and are appropriate targets for reducing the risk of specific multimorbidity groups in mid-life women.

## Introduction

As the ageing population continues to grow worldwide and the prevalence of chronic disease increases [1–3], especially in low and middle income countries as a result of the epidemiological transition, multimorbidity—the co-existence of multiple chronic diseases—has become an important public health issue. Of particular concern is the growing burden of multimorbidity among mid-aged adults [4, 5]. The individual, population and economic impact of individuals living for longer but with more co-existing diseases from a younger age highlights the need for a better understanding of the natural history of multimorbidity.

In particular, the manner in which chronic disease conditions cluster remains poorly understood, but has been increasingly studied in the past five years. A recent review identified 14 studies that aimed to identify patterns of associative multimorbidity, that is, non-random association between diseases [6]. However, just four of these studies were population-based [7–10], only two of which included individuals aged younger than 65 years [7, 10]. The remaining studies included selected study populations, with individuals identified from hospital admissions or outpatient clinics, Veterans networks or employee records [6], which limits representation of the general population. Despite variation in observed disease clusters across studies, three patterns did consistently emerge, reflecting the clustering of cardiovascular and metabolic syndrome-related diseases, mental health conditions and musculoskeletal disorders. Furthermore, as with most of the literature on multimorbidity to date, the majority of identified studies included adults aged over 65 years, and few distinguished between men and women. Given the marked public health burden of multimorbidity among working-age older adults, there is a need to better understand the epidemiology of multimorbidity within this younger age group. Furthermore, gender differences are likely to exist. In particular, the post-menopausal health trajectories specific to women support the need for the separate study of multimorbidity and its determinants among this particular demographic group.

Identification of multimorbidity patterns is useful for a number of reasons. These include the generation of hypotheses regarding common antecedents or disease pathways, improved prevention and management, and prediction of health-care use and adverse health outcomes. To date, little attention has been given to the determinants of multimorbidity in general [11]. Specifically, to our knowledge, no study has sought to examine the lifestyle and socioeconomic factors associated with subsequent multimorbidity patterns. This may shed further light on our understanding of why certain diseases cluster and may help inform the prevention of multimorbidity.

We therefore sought to ascertain patterns of associative multimorbidity in a cohort of mid-aged women and to identify the associations between lifestyle and socioeconomic factors at baseline with the subsequent development of these multimorbidity patterns.

## Methods

### Study setting

Participants were from the Australian Longitudinal Study on Women’s Health (ALSWH), a national population-based study of women born in 1921–26, 1946–51 and 1973–78. Women were randomly selected from the Medicare database, which covers all citizens and permanent residents of Australia, including refugees and immigrants. Women born in 1946–51 were surveyed using self-administered questionnaires in 1996 (survey 1, S1), 1998 (survey 2, S2), and every three years thereafter until 2010 (S6). Full details of recruitment and response rates are reported elsewhere [12]. In accordance with the Declaration of Helsinki, ethical approval for the ALSWH was obtained from the Universities of Newcastle and Queensland Research Ethics Committees and all participants gave informed consent to be included in the study.

### Study population

We included women from the 1946–51 cohort, which recruited 13,715 women aged 45–50 at S1, 12,338 (90%) of whom returned S2 and 10,011 (73%) returned S6. The prevalence of some chronic diseases and of multiple co-existing conditions reported in S1 was higher than in the subsequent survey, which may be due to a telescoping effect. At S1 women may also have reported on conditions that were historical, rather than chronic, such as pregnancy-related hypertension or gestational diabetes. We therefore relied on information reported at S2 on disease occurrence and treatment in the last two years to identify women with fewer than two reported diseases at baseline. For the purpose of this study we therefore included women free from multimorbidity at S2 who returned S6.

### Exposures

Lifestyle exposure variables were derived from information provided at S2. Smoking was classified as never, ex-smoker or current smoker. Body mass index (kg/m^2^) was computed as self-reported weight (kg)/height (m^2^), and subsequently categorised into underweight (<18.5 kg/m^2^), normal weight (18.5–24.9 kg/m^2^), overweight (25–29.9 kg/m^2^) or obese (≥ 30 kg/m^2^). At S2 physical activity was assessed using a modified (self-report) version of the Active Australia Physical Activity Survey.[12, 13] The women were asked to report frequency and total duration of walking, moderate, and vigorous intensity leisure time physical activity during the last week. A physical activity score in metabolic equivalent (MET) minutes per week was derived using the following formula: MET min/week = (walking minutes * 3.5 METs) + (moderate minutes * 4.0 METs) + (vigorous minutes * 7.5 METs). Physical activity was categorized as sedentary (0–39 MET min/week), low (40–599 MET min/week), moderate (600–1199 MET min/week) and high (≥ 1200 MET min/week). Alcohol intake was defined in light of the Australian National Health and Medical Research Council (NHMRC) guidelines with ‘Risky drinkers’ (15 to 28 drinks per week) and ‘High risk drinkers’ (more than 28 drinks per week) categorised accordingly.[14] Given the low frequency of high risk drinkers, the latter two groups were combined for analyses. For women identified as low risk by the NHMRC guidelines, we separately categorised women who reported that they drink only rarely (any alcohol consumption less than once a month) and non-drinkers, with the remainder classified as low-risk drinkers (up to 14 drinks per week).

Socioeconomic position (SEP) was determined using a range of measures collected at S1 (education and occupation) or S2 (manage on income and area-based deprivation). Education level was classified as high (university degree or diploma), middle (trade/apprenticeship or high school qualification(s)) or low (no formal qualifications) and own occupation was categorised as high (manager/professional/paraprofessional), middle (trade/administrative service) or low (manual worker). For completeness, a fourth category of women who reported having never worked or having an ‘other’ occupation was included. Women were also asked how well they managed on their income and could respond ‘easy’, ‘not bad’, ‘difficult sometimes’, ‘difficult all of the time’ or ‘impossible’. We categorised this variable into: easy/not bad; difficult sometimes; and difficult all of the time/impossible. Area of residence, classified as urban or rural/remote, was included as a covariate in the statistical modelling since women from rural/remote areas were deliberately over-sampled to ensure sufficient representation of women from these less populated areas.

### Multimorbidity outcome

We used information on symptoms and their severity as well as doctor-diagnosed diseases to cover as wide a range of conditions as possible, and to more accurately reflect the morbidity in the cohort. Occurrence of 18 chronic diseases and 13 symptoms at S6, as listed in Table 1, were ascertained through self-report. Women were asked if they had been diagnosed with or treated for each of the diseases in the past three years. They were also asked about the frequency of symptoms—‘Have you had any of the following problems in the last 12 months?’—and could respond: never; rarely; sometimes; or often. For these analyses, we dichotomised the responses to yes/no, with yes coded if the symptom was reported as occurring ‘often’. The exception was chest pain, which was considered present if women reported that this occurred ‘sometimes’ or ‘often’, due to the serious nature of chest pain. If women reported having experienced depression or anxiety/panic attacks ‘often’ in the past 12 months, this was incorporated into the depression and anxiety/nervous disorders disease variables.

**Table 1:** Prevalence of conditions at survey 6.

Condition	Prevalence at Survey 6 N (%)
Hypertension	1589 (32.5)
Other arthritis	1570 (32.1)
Joint stiffness/pain*	1019 (20.8)
Osteoarthritis	943 (19.3)
Back pain*	687 (14.0)
Asthma	639 (13.1)
Bronchitis/emphysema	549 (11.2)
Osteoporosis	484 (9.9)
Other cancer	467 (9.5)
Allergies*	457 (9.3)
Anxiety/nervous disorder	445 (9.1)
Vision problems*	406 (8.3)
Depression	361 (7.4)
Severe tiredness*	328 (6.7)
Diabetes	321 (6.6)
Chest pain*	301 (6.2)
Bowel problems*	287 (5.9)
Urinary problems*	257 (5.3)
Heart disease	252 (5.2)
Rheumatoid arthritis	255 (5.2)
Severe headache/migraine*	255 (5.2)
Hearing problems*	228 (4.7)
Impaired glucose tolerance	178 (3.6)
Poor memory*	177 (3.6)
Breast cancer	103 (2.1)
Chronic fatigue syndrome	67 (1.4)
Palpitations*	57 (1.2)
Stroke	52 (1.1)
Breathing difficulties*	55 (1.1)
Other psychiatric condition†	14 (0.3)
Cervical cancer	11 (0.2)

*Symptoms

^†^Other than depression or anxiety

### Statistical analysis

We performed the factor analysis using SAS version 9.4 and the regression analyses using Stata version 13.0

#### Factor analysis

To identify multimorbidity ‘patterns’ at S6, we performed exploratory factor analysis [15] to analyse correlations between conditions. We adopted factor analysis over other techniques because we were interested in how *conditions* (as opposed to *individuals*) group together. Factor analysis allowed us to identify associations between conditions, whilst allowing these conditions to cross-load (i.e. belong to more than one factor or pattern). Since each condition was coded as a binary variable, we computed tetrachoric correlations matrix between all conditions [16]. This was done by using the macro “Polychor” in SAS 9.4. We then carried out factor analysis on the correlation matrix by applying “method = Principal” option in Proc Factor. The number of factors identified was based on their interpretability, having an eigenvalue greater than one, and the shape of the scree plot [15]. We used a varimax rotation of factor loading matrices, with each resulting factor loading representing the strength of association between the condition and the latent factor. We obtained factor scores for each participant standardised to a mean of 0 and standard deviation (SD) of 1. For ease of interpretation of the results we created tertiles of factor scores, with the lowest third representing a low factor score and the highest third representing a high factor score (i.e. women with a high score for the multimorbidity pattern).

#### Association of risk factors with multimorbidity patterns

We summarised the frequency of each lifestyle factor and SEP measure by morbidity pattern.

Given that the factor scores were not normally distributed, we used the top 25^th^ percentile as a cut point to create a dichotomous variable for each factor (i.e. a high versus low score) and used logistic regression to relate lifestyle and SEP factors to subsequent multimorbidity patterns. We therefore obtained unadjusted and adjusted odds ratios (ORs) with accompanying confidence intervals (CIs) for the effect of each determinant on the odds of having a high score for each multimorbidity pattern. Adjusted analyses controlled for age, SEP, area of residence and all included lifestyle factors.

The manuscript was prepared in accordance with the STROBE guidelines.

## Results

Of the 10,011 women who returned S6, 5388 reported fewer than two diseases at S2. Of these, there was complete data on conditions at S6 among 4896 (91%) women. We included these 4896 women, with a mean age of 49.5 (±1.5) at S2 and 61.5 (± 1.5) years at S6, in the factor analysis. The most common condition was hypertension (32.5%) and the least common was cervical cancer (0.2%; Table 1).

### Multimorbidity patterns

We identified five factors, or multimorbidity patterns, that explained 28.5% of the variance (Table 2); ‘psychosomatic’ (characterised by anxiety, depression and somatic symptoms including severe tiredness, severe headache/migraine bowel problems and palpitations), ‘musculoskeletal’ (primarily characterised by arthritis, joint and back pain), ‘cardiometabolic’ (characterised by cardiovascular disease, diabetes and impaired glucose tolerance), ‘cancer’ and ‘respiratory’ (characterised mainly by asthma, bronchitis/emphysema and breathing difficulties) (Table 2). Given the inherent subjectivity of analytic decisions made during factor analysis, we have also provided information on the four and five-factor solutions. A four-factor solution explained 24.4% of the variability, with similar factors identified as in the 5-factor solution, with the exception that it did not include the respiratory factor. A 6-factor solution, which explained 32.3% of the variability, with an eigenvalue of 1.18, identified similar patterns as in the 5-factor analysis. However, instead of identifying one factor characterised by ‘psychosomatic disease’, two factors were identified, which were characterised by depression, anxiety and other psychiatric illness in one factor and somatic symptoms in the other. The scree plot indicated an ‘elbow’ (i.e. a flattening of the eigenvalue) at six factors. We chose a five-factor solution after careful consideration of the eigenvalues and the scree plot (bearing in mind the general rule that components should be retained before the first point that starts the flat line trend), and for the parsimony and interpretability of the different factor solutions.

**Table 2:** Factor loadings^a^ for chronic diseases and symptoms at survey 6.

	Factor/pattern				
	Psychosomatic	Musculoskeletal	Cardiometabolic	Cancer	Respiratory
Anxiety	**0.56**	-0.13	0.16	-0.01	-0.03
Depression	**0.55**	-0.10	0.18	0.06	0
Severe tiredness^b^	**0.53**	0.17	0.05	0.10	-0.06
Poor memory^b^	**0.42**	0.03	0	0.01	-0.05
Severe headache^b^	**0.37**	0.03	-0.02	-0.05	-0.01
Chest pain^b^	**0.36**	0.03	0.09	-0.07	0.18
Vision problems^b^	**0.35**	0.11	-0.04	0.07	0.02
Bowel problems^b^	**0.35**	0.11	-0.06	0.01	0.09
Palpitations^b^	**0.30**	-0.01	0.06	-0.06	0.02
Hearing problems^b^	0.24	0.07	-0.06	0.04	0.07
Urinary problems^b^	0.24	0.12	-0.03	-0.01	0.14
Joint stiffness/pain^b^	**0.28**	**0.70**	-0.02	-0.03	-0.04
Other arthritis	0.05	**0.65**	0.08	-0.03	0.05
Osteoarthritis	0.05	**0.64**	0.07	0	0.04
Back pain^b^	0.34	**0.55**	-0.02	-0.03	-0.02
Rheumatoid arthritis	-0.05	**0.37**	**0.28**	0.01	0.20
Osteoporosis	-0.04	**0.24**	0.05	0.10	0.19
Impaired glucose tolerance	-0.03	0.08	**0.62**	-0.06	-0.11
Diabetes	-0.03	0.06	**0.61**	0.03	-0.15
Stroke	-0.01	0.01	**0.51**	0.13	0.23
Chronic fatigue syndrome	0.11	0.03	**0.41**	0.17	0.18
Heart disease	0.09	0.02	**0.38**	0.03	**0.32**
Hypertension	0.02	0.20	**0.37**	-0.09	-0.15
Other psychiatric disorders	0.15	-0.12	**0.25**	0.15	0.09
Other cancers	-0.03	0.03	0.04	**0.81**	-0.02
Breast cancer	-0.01	0.05	-0.05	**0.79**	-0.07
Cervical cancer	0.02	-0.06	0.15	**0.39**	0.06
Asthma	-0.04	0.01	0.09	0.02	**0.57**
Bronchitis/emphysema	0.04	0.05	0.07	0.01	**0.55**
Breathing difficulties^b^	0.10	0.01	-0.08	-0.04	**0.53**
Allergies^b^	0.19	0.11	-0.14	0	**0.26**
*Eigenvalue*	*2*.*83*	*1*.*79*	*1*.*53*	*1*.*41*	*1*.*27*

^a^Factor loadings indicate the strength of association between each variable and each factor, with a factor loading of ≤0.2 (non-bold loadings) generally considered to be weak

^b^Symptoms

### Risk factors for multimorbidity patterns

Table 3 summarises the distribution of lifestyle and SEP factors according to multimorbidity factor score.

**Table 3:** Baseline characteristics by multimorbidity factor score (low or high) at survey 6.

	Multimorbidity pattern (N = 4896)									
	Psychosomatic (%)		Musculoskeletal (%)		Cardiometabolic (%)		Cancer (%)		Respiratory (%)	
Baseline characteristic	Low	High	Low	High	Low	High	Low	High	Low	High
BMI (kg/m^2^)										
Underweight (<18.5)	45 (1.3)	11 (19.6)	50 (1.5)	6 (0.5)	43 (1.3)	13 (23.2)	38 (1.1)	18 (1.6)	39 (1.2)	17 (30.4)
Normal (18.5–24.9)	1747 (51.7)	555 (46.7)	1835 (54.6)	467 (41.2)	1804 (55.7)	498 (39.7)	1635 (48.7)	667 (58.7)	1727 (51.2)	575 (51.3)
Overweight (25.0–29.9)	1073 (31.8)	370 (33.1)	1046 (31.1)	397 (35.0)	994 (30.7)	449 (35.8)	1117 (33.3)	326 (28.7)	1090 (32.3)	353 (31.5)
Obese (≥30)	512 (15.2)	181 (16.2)	429 (12.8)	264 (23.3)	400 (12.3)	293 (23.4)	568 (16.9)	125 (11.0)	517 (15.3)	176 (15.7)
Physical activity										
High	1094 (31.3)	326 (28.5)	1110 (31.9)	310 (26.8)	1041 (31.1)	379 (29.3)	1083 (31.1)	337 (29.1)	1039 (29.9)	1420 (30.6)
Moderate	828 (23.7)	255 (22.3)	828 (23.8)	255 (22.0)	802 (24.0)	281 (21.7)	793 (22.8)	290 (25.0)	823 (23.7)	1083 (23.3)
Low	1070 (30.6)	347 (30.3)	1040 (29.9)	377 (32.6)	1009 (30.2)	408 (31.5)	1086 (31.2)	331 (28.6)	1075 (30.9)	1417 (30.5)
Nil/sedentary	502 (14.4)	218 (19.0)	504 (14.5)	216 (18.7)	493 (14.7)	227 (17.5)	520 (14.9)	200 (17.3)	541 (15.6)	720 (15.5)
Smoking										
Never	2123 (59.5)	681 (57.7)	2134 (60.0)	670 (56.2)	2009 (58.8)	795 (59.5)	2128 (59.7)	676 (57.0)	2167 (60.8)	637 (53.6)
Ex	965 (27.0)	304 (25.7)	940 (26.4)	329 (27.6)	923 (27.0)	346 (25.9)	946 (26.5)	323 (27.2)	941 (26.4)	328 (27.6)
Current	482 (13.5)	196 (16.6)	484 (13.6)	194 (16.3)	483 (14.1)	195 (14.6)	490 (13.8)	188 (15.8)	454 (12.8)	224 (18.8)
Alcohol intake										
Low risk	2091 (58.6)	642 (54.3)	2062 (58.1)	671 (56.0)	2033 (59.6)	700 (52.4)	2062 (57.9)	671 (56.6)	2054 (57.7)	679 (57.1)
Non-drinker	1309 (36.7)	476 (40.3)	1318 (37.1)	467 (39.0)	1219 (35.7)	566 (42.4)	1326 (37.2)	459 (38.7)	1334 (37.5)	451 (37.9)
Risky/high-risk	166 (4.7)	64 (5.4)	170 (4.8)	60 (5.0)	161 (4.7)	69 (5.2)	174 (4.9)	56 (4.7)	170 (4.8)	60 (5.0)
Education										
High	1287 (35.3)	367 (30.2)	1294 (35.5)	360 (29.7)	1254 (35.9)	400 (29.4)	1200 (33.0)	454 (37.3)	1224 (33.6)	430 (35.3)
Middle	1910 (52.4)	638 (52.5)	1899 (52.1)	649 (53.5)	1809 (51.7)	739 (54.3)	1948 (53.5)	600 (49.3)	1933 (53.1)	615 (50.5)
Low	446 (12.2)	210 (17.3)	452 (12.4)	204 (16.8)	433 (12.4)	223 (16.4)	494 (13.6)	162 (13.3)	483 (13.3)	173 (14.2)
Occupation										
High	1508 (42.4)	439 (37.5)	1478 (41.6)	469 (40.1)	1453 (42.5)	494 (37.8)	1412 (39.9)	535 (45.3)	1476 (41.5)	471 (40.3)
Middle	1545 (43.5)	544 (46.5)	1560 (43.9)	529 (45.2)	1481 (43.3)	608 (46.5)	1604 (45.3)	485 (41.1)	1559 (43.9)	530 (25.4)
Low	416 (11.7)	161 (13.8)	430 (12.1)	147 (12.6)	411 (12.0)	166 (12.7)	445 (12.6)	132 (11.2)	430 (12.1)	147 (12.6)
Never paid work/other	85 (2.4)	26 (2.2)	85 (2.4)	26 (2.2)	72 (2.1)	39 (3.0)	82 (2.3)	29 (2.5)	89 (2.5)	22 (1.9)
Ability to manage on income										
Easy/not bad	2309 (65.0)	667 (56.4)	2270 (64.0)	706 (59.3)	2198 (64.6)	778 (58.5)	2225 (62.6)	751 (63.5)	2238 (63.1)	738 (62.2)
Sometimes difficult	930 (26.2)	346 (29.3)	939 (26.5)	337 (28.3)	882 (25.9)	394 (29.6)	971 (27.3)	305 (25.8)	939 (26.5)	337 (28.4)
Impossible/difficult always	314 (8.8)	169 (14.3)	336 (9.5)	147 (12.4)	324 (9.5)	159 (12.0)	356 (10.0)	127 (10.7)	372 (10.5)	111 (9.4)

In unadjusted analyses, overweight and obesity were associated with significantly increased odds of a high score for the musculoskeletal and cardiometabolic patterns, as was increasing BMI (Table 4). Physical inactivity was associated with increased odds of a high score for all patterns, except the respiratory pattern, whilst current smoking was associated with increased odds of a high score for all patterns except for the cardiometabolic pattern. No alcohol intake was associated with increased odds of high psychosomatic and cardiometabolic pattern scores.

**Table 4:** Unadjusted ORs of the association between characteristics and a high versus low multimorbidity pattern score.

Characteristics	Multimorbidity pattern^a^				
	Psychosomatic	Musculoskeletal	Cardiometabolic	Cancer	Respiratory
	OR (95% CI)	OR (95% CI)	OR (95% CI)	OR (95% CI)	OR (95% CI)
BMI (kg/m^2^)^b^					
Underweight (<18.5)	0.77 (0.40, 1.50)	0.47 (0.20, 1.11)	1.10 (0.58, 2.05)	1.16 (0.66, 2.05)	1.31 (0.73, 2.33)
Normal (18.5–24.9)	1.00	1.00	1.00	1.00	1.00
Overweight (25.0–29.9)	1.09 (0.93, 1.26)	1.49 (1.28, 1.74)	1.64 (1.41, 1.90)	0.72 (0.61, 0.83)	0.97 (0.83, 1.13)
Obese (≥30)	1.11 (0.92, 1.35)	2.42 (2.01, 2.91)	2.65 (2.22, 3.18)	0.54 (0.44, 0.67)	1.02 (0.84, 1.24)
BMI (kg/m^2^)^b^^,^^c^	1.01 (1.00, 1.02)	1.08 (1.07, 1.09)	1.08 (1.07, 1.10)	0.95 (0.93, 0.96)	0.99 (0.98, 1.01)
Physical activity					
High	1.00	1.00	1.00	1.00	1.00
Moderate	1.03 (0.86, 1.25)	1.10 (0.91, 1.33)	0.96 (0.80, 1.15)	1.18 (0.98, 1.41)	0.86 (0.72, 1.03)
Low	1.09 (0.92, 1.29)	1.30 (1.09, 1.54)	1.11 (0.94, 1.31)	0.98 (0.82, 1.17)	0.87 (0.73, 1.03)
Nil/sedentary	1.46 (1.19, 1.78)	1.53 (1.25, 1.88)	1.26 (1.04, 1.54)	1.24 (1.01, 1.52)	0.90 (0.73, 1.11)
Smoking					
Never	1.00	1.00	1.00	1.00	1.00
Ex	0.98 (0.84, 1.15)	1.11 (0.96, 1.30)	0.95 (0.82, 1.10)	1.07 (0.92, 1.25)	1.19 (1.02, 1.38)
Current	1.27 (1.05, 1.53)	1.28 (1.06, 1.04)	1.02 (0.85, 1.23)	1.21 (1.00, 1.46)	1.68 (1.40, 2.02)
Alcohol intake					
Low risk	1.00	1.00	1.00	1.00	1.00
Non-drinker	1.18 (1.03, 1.36)	1.09 (0.95, 1.25)	1.35 (1.18, 1.54)	1.06 (0.93, 1.22)	1.02 (0.89, 1.17)
Risky/high-risk	1.26 (0.93, 1.70)	1.08 (0.80, 1.47)	1.24 (0.93, 1.67)	0.99 (0.72, 1.35)	1.07 (0.79, 1.45)
Education					
High	1.00	1.00	1.00	1.00	1.00
Middle	1.17 (1.01, 1.36)	1.23 (1.06, 1.42)	1.28 (1.11, 1.48)	0.81 (0.71, 0.94)	0.91 (0.79, 1.04)
Low	1.65 (1.35, 2.02)	1.62 (1.33, 1.99)	1.61 (1.33, 1.97)	0.87 (0.70, 1.07)	1.02 (0.83, 1.25)
Occupation					
High	1.00	1.00	1.00	1.00	1.00
Middle	1.21 (1.05, 1.40)	1.07 (0.93, 1.23)	1.21 (1.05, 1.39)	0.80 (0.69, 0.92)	1.07 (0.92, 1.23)
Low	1.33 (1.08, 1.64)	1.08 (0.87, 1.33)	1.19 (0.97, 1.46)	0.78 (0.63, 0.97)	1.07 (0.86, 1.33)
Never paid work/other	1.05 (0.67, 1.65)	0.96 (0.61, 1.51)	1.59 (1.06, 2.39)	0.93 (0.61, 1.44)	0.77 (0.48, 1.25)
Ability to manage on income					
Easy/not bad	1.00	1.00	1.00	1.00	1.00
Sometimes difficult	1.29 (1.11, 1.50)	1.53 (0.99, 1.34)	1.26 (1.09, 1.46)	0.93 (0.80, 1.08)	1.09 (0.94, 1.26)
Impossible/difficult always	1.86 (1.52, 2.29)	1.41 (1.14, 1.74)	1.39 (1.13, 1.70)	1.06 (0.85, 1.32)	0.90 (0.72, 1.14)

^a^Patterns identified at survey 6 (N = 4896)

^b^We performed two models, one with BMI as a categorical variable and one with BMI as a continuous variable

^c^The odds ratio represent the increase or decrease in odds of the outcome relative to the reference group, per unit increase in BMI

BMI = body mass index; CI = confidence interval; OR = odds ratio;

Low education and difficulties managing on income were also associated with increased odds of high scores for the psychosomatic, musculoskeletal and cardiometabolic patterns (Table 4).

In adjusted analyses, increasing BMI remained associated with increased odds of the musculoskeletal and cardiometabolic patterns, with a unit increase in BMI associated with a 7% increase in odds for each of these patterns. Being overweight was associated with a 1.5 fold increased odds of a high score for the musculoskeletal and cardiometabolic patterns (OR 1.45, 95% CI 1.23 to 1.70 and OR 1.53, 95% CI 1.31 to 1.79 respectively; Table 5), whilst obesity was associated with a more than two-fold increased odds of a high score for these patterns (OR 2.14, 95% CI 1.75 to 2.60 and OR 2.46, 95% CI 2.02 to 2.98). In contrast to the other patterns, overweight and obesity were associated with decreased odds of having a high respiratory factor score (OR 0.74, 95% CI 0.63 to 0.87 and 0.50, 95% CI 0.39 to 0.62, respectively). Physical inactivity increased the odds of a high score for the psychosomatic pattern by 41% (OR 1.41, 95% CI 1.13 to 1.76), the musculoskeletal pattern by 39% (OR 1.39, 95% CI 1.11 to 1.74) and the cancer pattern by 35% (OR 1.35, 95% CI 1.08 to 1.69). The associations for current smoking observed in unadjusted analyses remained significant in fully adjusted analyses for the musculoskeletal and respiratory patterns (OR 1.24, 95% CI 1.01 to 1.54 and 1.74, 95% CI 1.42 to 2.13, respectively; Table 5). Ex-smoking also significantly increased the odds of a high respiratory factors score in the adjusted model (OR 1.23, 95% CI 1.04 to 1.45). The association between no alcohol intake and increased odds of having a high score for the cardiometabolic pattern persisted in adjusted analyses (OR 1.18, 95% CI 1.02 to 1.37).

**Table 5:** Adjusted ORs^a^ of the association between characteristics and a high versus low multimorbidity pattern score.

Characteristics	Multimorbidity pattern				
	Psychosomatic	Musculoskeletal	Cardiometabolic	Cancer	Respiratory
	OR (95% CI)	OR (95% CI)	OR (95% CI)	OR (95% CI)	OR (95% CI)
BMI (kg/m^2^)^b^					
Underweight (<18.5)	0.68 (0.34, 1.38)	0.50 (0.21, 1.17)	1.07 (0.55, 2.06)	1.02 (0.56, 1.87)	1.20 (0.65, 2.22)
Normal (18.5–24.9)	1.00	1.00	1.00	1.00	1.00
Overweight (25.0–29.9)	1.04 (0.88, 1.22)	1.45 (1.23, 1.70)	1.53 (1.31, 1.79)	0.74 (0.63, 0.87)	0.96 (0.82, 1.13)
Obese (≥30)	1.01 (0.82, 1.25)	2.14 (1.75, 2.60)	2.46 (2.02, 2.98)	0.50 (0.39, 0.62)	1.09 (0.89, 1.34)
BMI (kg/m^2^)^b^^,^^c^	1.00 (0.99, 1.02)	1.07 (1.06, 1.09)	1.07 (1.06, 1.09)	0.95 (0.93, 0.96)	1.00 (0.98, 1.01)
Physical activity					
High	1.00	1.00	1.00	1.00	1.00
Moderate	1.10 (0.90, 1.34)	1.10 (0.90, 1.35)	0.93 (0.76, 1.12)	1.20 (0.99, 1.45)	0.90 (0.74, 1.09)
Low	1.06 (0.88, 1.28)	1.23 (1.02, 1.48)	1.02 (0.86, 1.23)	1.06 (0.88, 1.28)	0.89 (0.74, 1.06)
Nil/sedentary	1.41 (1.13, 1.76)	1.39 (1.11, 1.74)	1.08 (0.87, 1.35)	1.35 (1.08, 1.69)	0.99 (0.79, 1.24)
Smoking					
Never	1.00	1.00	1.00	1.00	1.00
Ex	1.05 (0.89, 1.25)	1.06 (0.90, 1.26)	0.98 (0.83, 1.16)	1.10 (0.93, 1.30)	1.23 (1.04, 1.45)
Current	1.23 (0.99, 1.51)	1.24 (1.01, 1.54)	1.02 (0.82, 1.25)	1.21 (0.98, 1.48)	1.74 (1.42, 2.13)
Alcohol intake					
Low risk	1.00	1.00	1.00	1.00	1.00
Non-drinker	1.10 (0.94, 1.29)	0.96 (0.82, 1.12)	1.18 (1.02, 1.37)	1.16 (0.99, 1.35)	1.03 (0.88, 1.20)
Risky/high-risk	1.17 (0.85, 1.63)	1.00 (0.72, 1.40)	1.18 (0.85, 1.63)	1.02 (0.73, 1.42)	0.97 (0.69, 1.34)
Education					
High	1.00	1.00	1.00	1.00	1.00
Middle	1.02 (0.85, 1.23)	1.20 (0.99, 1.44)	1.17 (0.98, 1.41)	0.96 (0.80, 1.15)	0.81 (0.67, 1.97)
Low	1.34 (1.03, 1.75)	1.43 (1.10, 1.87)	1.29 (1.00, 1.68)	1.02 (0.78, 1.34)	0.94 (0.72, 1.23)
Occupation					
High	1.00	1.00	1.00	1.00	1.00
Middle	1.15 (0.96, 1.38)	0.97 (0.81, 1.16)	1.02 (0.85, 1.21)	0.85 (0.71, 1.01)	1.15 (0.96, 1.38)
Low	1.08 (0.82, 1.40)	0.89 (0.68, 1.17)	0.88 (0.68, 1.14)	0.78 (0.60, 1.03)	1.23 (0.95, 1.60)
Never paid work/other	0.83 (0.49, 1.42)	0.83 (0.50, 1.39)	1.50 (0.95, 2.38)	1.03 (0.64, 1.68)	0.74 (0.42, 1.30)
Ability to manage on income					
Easy/not bad	1.00	1.00	1.00	1.00	1.00
Sometimes difficult	1.31 (1.11, 1.54)	1.04 (0.88, 1.23)	1.17 (1.00, 1.37)	1.01 (0.85, 1.18)	1.04 (0.89, 1.23)
Impossible/difficult always	1.73 (1.37, 2.18)	1.38 (1.09, 1.75)	1.23 (0.97, 1.56)	1.17 (0.92, 1.50)	0.95 (0.74, 1.23)

^a^Odds ratios adjusted for all dependent variables given in the table, plus age and area of residence

^b^We performed two separate models; one that included BMI as a categorical variable and one that included BMI as a continuous variable. The odds ratios for the other exposure variables are adjusted for BMI included as a categorical variable

^c^The odds ratio represent the increase or decrease in odds of the outcome relative to the reference group, per unit increase in BMI

BMI = body mass index; CI = confidence interval; OR = odds ratio;

After controlling for all lifestyle and other SEP factors, a low education level was significantly associated with increased odds of the psychosomatic and musculoskeletal patterns (OR 1.34, 95% CI 1.03 to 1.75 and OR 1.43, 95% CI 1.10 to 1.87, respectively; Table 5), but not the cardiometabolic pattern. Similarly, difficulty managing on income remained associated with increased odds of a high score for the psychosomatic and musculoskeletal factors (OR 1.73, 95% CI 1.37 to 2.18 and OR 1.38, 95% CI 1.09 to 1.75, respectively) in adjusted analyses, whilst the association for the cardiometabolic pattern was borderline significant. There was no association between occupation and any of the observed multimorbidity patterns.

## Discussion

We identified five multimorbidity patterns—psychosomatic, musculoskeletal, cardiometabolic, cancer and respiratory—in a cohort of mid-aged women. Our study is one of just a few population-based studies in which multimorbidity patterns have been examined and one of even fewer to have investigated this in a younger age-group. To our knowledge, this is the first study to relate baseline characteristics to multimorbidity patterns. Some patterns were differentially associated with baseline characteristics, whereas others shared common risk factors, such as physical activity, overweight/obesity and SEP, as measured by education and ability to manage on income. Importantly, our findings identify which modifiable risk factors are associated with increased risk of multiple patterns and thus provide evidence for where best to invest primary prevention approaches for reducing risk of these multimorbidity patterns. Our results also identify specific risk factors which appear to be particularly important for certain patterns. For instance, physical inactivity, but not overweight/obesity, along with education, appears to be especially important in terms of being associated with increased risk of the patterns characterised partly by poor mental health.

Our identification of five multimorbidity patterns is in keeping with the results from other studies that investigated patterns of associative multimorbidity using the same statistical approach, which generally identified three to six patterns [8, 10, 17–19]. Although a recent systematic review identified considerable variation in the nature of disease clusters across 14 relevant studies, they did find some similarities, with cardiometabolic, mental health and musculoskeletal-related patterns consistently emerging from many studies [6], including from those that used factor analysis, as in our study [8, 10, 18, 19]. The patterns identified in our cohort are similar to those identified in a study that used the same analytical approach and stratified by gender and age. This study identified three patterns—cardiometabolic, mechanical and depressive—in women aged 45–64 years [10]. Differences in the number and nature of multimorbidity patterns across studies likely reflects differences in study populations, method of ascertaining chronic conditions, the number of conditions included and the analytic approach employed.

In the studies that included obesity as a chronic disease in their analyses, obesity tended to cluster with cardiometabolic patterns [8, 10, 18, 19] and with musculoskeletal disorders [10, 18, 20, 21]. Since we were interested in examining modifiable lifestyle factors associated with subsequent multimorbidity patterns, we included BMI as an exposure variable in our analyses, as opposed to a condition. Our findings on the association between being overweight or obese and subsequent development of the cardiometabolic pattern and musculoskeletal patterns suggest that overweight/obesity is a key risk factor for the development of particular disease clusters, thus accounting for the co-existence of obesity with these chronic conditions.

Some of the observed associations between lifestyle factors and specific multimorbidity patterns are in line with what we would expect, based on the clear understanding of associations between certain lifestyle factors and various chronic diseases. For example, the significant association between smoking and an increased risk of the respiratory pattern is consistent with the established association between smoking and respiratory symptoms such asthma, bronchitis and breathing difficulties [22, 23]. Our study identified antecedents common to multiple multimorbidity patterns. Physical activity and BMI in particular appear to be important factors associated with the development of a number of multimorbidity patterns, with the associations for BMI especially being very strong. The prospective association between overweight/obesity and the cardiometabolic pattern is in keeping with the known associations between excess body weight and conditions such as hypertension, diabetes and heart disease [24]. Not only does excess weight exert a direct effect, being a common determinant of major chronic conditions, it also has an indirect effect due to the relationship between conditions (e.g. type 2 diabetes is associated with subsequent increased risk of cardiovascular disease). There is a well-established body of evidence for obesity being a risk factor for various musculoskeletal disorders [25]. Our finding that obesity is strongly associated with an increased risk of the musculoskeletal pattern is consistent with this evidence. In addition, our results suggest that being overweight also markedly increases the risk of musculoskeletal conditions and appears to be an even stronger risk factor for the musculoskeletal than the cardiometabolic pattern. The negative association between overweight/obesity and odds of the cancer pattern is an interesting finding. Whilst the evidence for an association between higher BMI and an increased risk of some cancers is substantial, the evidence for other cancers, such as ovarian cancer, is inconclusive [26]. The ALSWH survey asked women about the occurrence of specific cancers, including skin cancer, and then asked about ‘other cancers’ in general. Whilst we didn’t include skin cancer as one of the disease outcomes in our factor analyses, the cohort still included women with skin cancer, which may have impacted our findings and may partly explain the observed inverse association between overweight/obesity and the cancer pattern. Higher BMI has been found to be associated with a decreased risk of non-melanoma skin cancer, possibly due to lower sun exposure [27].

There is a growing body of evidence linking physical activity with musculoskeletal fitness, bone loss and risk of chronic disease such as arthritis and osteoporosis [28]. The observed association between little or no physical activity and increased risk of the musculoskeletal pattern in our study contributes further to this existing research. The association between physical activity and cancer in women is perhaps less well understood, with the strongest evidence supporting a role for physical activity in reducing breast cancer [29]. Our findings provide possible evidence for a role of physical activity in reducing the development of a multimorbidity pattern characterised by cancer.

Although there is a growing body of evidence linking increased physical activity with reduced risk of depression [30], the evidence for other measures of mental well-being is more limited. Whilst there is considerable evidence for a cross-sectional association between physical health and mental well-being, there is less prospective or interventional evidence supporting a role for physical activity in the prevention of poor mental health [31]. Our study makes an important contribution to this research area by highlighting the strong association between low physical activity and development of a psychosomatic multimorbidity pattern. These findings complement previous cross-sectional analyses of the same cohort of women which found an association between physical activity and mental well-being as measured by the mental health component of the Medical Outcomes Study Short Form-36 [32]. Our findings suggest that physical inactivity may be just as important, if not more so, in reducing risk of multimorbidity patterns characterised partly by mental health as much as physical disease.

Interestingly our study did not identify any significant associations between alcohol intake and any of the observed multimorbidity patterns, other than the positive association between no alcohol intake and risk of the cardiometabolic pattern. The latter is in keeping with the observed j-shaped association between alcohol intake and cardiovascular disease risk [33]. We were probably under-powered to detect a significant association between high-risk alcohol intake and the cardiometabolic pattern, given the low numbers of high-risk drinkers in this cohort of mid-aged women.

Socioeconomic gradients in physical health and some measures of mental health are well documented [34, 35]. The significant associations between education and the psychosomatic and musculoskeletal patterns are consistent with this. The association between education and the cardiometabolic pattern was however not quite statistically significant. Difficulty managing on income was markedly associated with the psychosomatic pattern, and complements previous research on the association between economic circumstances and common mental disorders [36].

Our study has various strengths. The use of factor analysis to identify morbidity patterns has a number of benefits: it does not rely on pre-conceived assumptions as to how particular conditions cluster together; it allows conditions to cross-load on multiple factors; and it facilitates a better understanding of how *conditions* (as opposed to *individuals*) naturally group together. We also used information collected on symptoms reported as being severe, as well as doctor-diagnosed disease, to cover as wide a range of conditions as possible, and to more accurately reflect the existing morbidity that women live with. There is debate in the literature regarding how to define multimorbidity and the need for a more holistic definition that is not restricted by a disease-focused medical label, and which encompasses wellbeing and severity of problems that people face [37]. These impairments can have a marked effect on quality of life, disability, health-care use and so on. It therefore seems appropriate to incorporate severe symptoms of morbidity, irrespective of whether those symptoms have been attributed to a particular disease or not. In general, as expected, symptoms tended to cluster with related diseases. Being a prospective study, we were able to examine the associations between risk factors at baseline and subsequent multimorbidity patterns, thereby minimising the introduction of reverse causality, whereby the development of multiple chronic conditions might in turn impact on lifestyle behaviour, such as the ability to exercise, or impact on employment and financial hardship. Finally, this is, to our knowledge, the first study to report on the association between lifestyle and socioeconomic factors and multimorbidity patterns, making a novel contribution to the increasingly important area of multimorbidity research.

Our study does have some limitations. The conditions used in the factor analysis were based on self-report, which may have introduced some errors. However, studies have validated self-report of various chronic diseases, with the most prevalent diseases in our study considered to have good self-report validity within this age-group [38]. Although in an older age group, a recent validation study of self-reported chronic conditions in multimorbid patients also suggests that there is moderate to good validity for many of the diseases included in our study [39]. Our list of included conditions is also not exhaustive and did not include a number of chronic diseases such as liver or kidney disease. The investigators did however try to focus on the most common chronic conditions when collecting these data. We also included women free from multimorbidity (i.e. with fewer than two conditions) at baseline, which means that some women were not completely disease-free at baseline. It is therefore possible that some reverse causation could have been introduced, through the presence of a particular disease affecting lifestyle or SEP. If presence of a particular disease had a positive effect on health behaviour but also contributed to the development of multimorbidity then we may have underestimated lifestyle-multimorbidity pattern associations. Conversely, if disease presence led to reduction in healthy behaviour, and also contributed to the development of multimorbidity then we may have over-estimated observed associations. Lifestyle factors were self-reported and thus susceptible to measurement error. However, self-reported height and weight have been shown to be valid for calculating BMI in this cohort [40], whilst the physical activity questionnaire also has measurement properties which compare favourably to those of other commonly used physical activity measures [41]. Relying on self-reported smoking and alcohol intake may have led to some measurement error, however these behaviours would tend to be under- rather than over-reported, which would likely lead to under- rather than over-estimation of effect estimates. Finally, our study included women only and so findings cannot be extrapolated to men. However, among the few studies that have analysed disease clusters by age and sex, there does appear to be key differences in the nature or prevalence of disease clusters between men and women [7, 9, 10, 19] and between mid-age and older people [7, 10]. Multimorbidity patterns are likely to differ by age and gender, and we might expect the determinants of these patterns, or the magnitude of their effect, to also differ. For instance, multimorbidity patterns and their determinants in post-menopausal working age women are likely to differ from those in men or indeed older women. Thus, separate study of, or stratification by, gender and age-groups is necessary.

### Conclusions

In conclusion, our study contributes to understanding the nature of multimorbidity among mid-aged women by identifying which conditions group together and which lifestyle and socioeconomic factors are potentially involved in the aetiology of these multimorbidity patterns. In terms of preventive approaches among mid-aged women, improving physical activity levels and reducing the proportion of women who are overweight or obese may be the most appropriate approach to reducing the risk of these disease groups.
